# Intravenous functional gene transfer throughout the brain of non-human primates using AAV

**DOI:** 10.21203/rs.3.rs-1370972/v1

**Published:** 2023-01-13

**Authors:** Miguel R. Chuapoco, Nicholas C. Flytzanis, Nick Goeden, J. Christopher Octeau, Kristina M. Roxas, Ken Y. Chan, Jon Scherrer, Janet Winchester, Roy J. Blackburn, Lillian J. Campos, Kwun Nok Mimi Man, Junqing Sun, Xinhong Chen, Arthur Lefevre, Vikram Pal Singh, Cynthia M. Arokiaraj, Timothy F. Shaya, Julia Vendemiatti, Min J. Jang, John Mich, Yeme Bishaw, Bryan Gore, Victoria Omstead, Naz Taskin, Natalie Weed, Jonathan Ting, Cory T. Miller, Benjamin E. Deverman, James Pickel, Lin Tian, Andrew S. Fox, Viviana Gradinaru

**Affiliations:** a Division of Biology and Biological Engineering, California Institute of Technology, Pasadena, CA 91125, USA; b Capsida Biotherapeutics, Thousand Oaks, CA 91320, USA; c Department of Psychology and the California National Primate Research Center, University of California-Davis, Davis, CA 95616, USA; d Cortical Systems and Behavior Laboratory, University of California-San Diego, La Jolla, CA 92039, USA; e Allen Institute for Brain Science, Seattle, WA, 98109, USA; f National Institute of Mental Health, National Institutes of Health, Bethesda, MD 20892 USA; g Aligning Science Across Parkinson’s (ASAP) Collaborative Research Network, Chevy Chase, MD 20815; h Present address: Capsida Biotherapeutics, Thousand Oaks, CA 91320, USA; i Present address: Stanley Center for Psychiatric Research, Broad Institute of MIT and Harvard, Massachusetts Institute of Technology, Cambridge, MA 02142, USA

## Abstract

Adeno-associated viruses (AAVs) promise robust gene delivery to the brain through non-invasive, intravenous delivery. However, unlike in rodents, few neurotropic AAVs efficiently cross the blood-brain barrier in non-human primates (NHPs). Here we describe AAV.CAP-Mac, an engineered variant identified by screening in adult marmosets and newborn macaques with improved efficiency in the brain of multiple NHP species: marmoset, rhesus macaque, and green monkey. CAP-Mac is neuron-biased in infant Old World primates, exhibits broad tropism in adult rhesus macaques, and is vasculature-biased in adult marmosets. We demonstrate applications of a single, intravenous dose of CAP-Mac to deliver (1) functional GCaMP for *ex vivo* calcium imaging across multiple brain areas, and (2) a cocktail of fluorescent reporters for Brainbow-like labeling throughout the macaque brain, circumventing the need for germline manipulations in Old World primates. Given its capabilities for systemic gene transfer in NHPs, CAP-Mac promises to help unlock non-invasive access to the brain.

Adeno-associated viruses (AAVs) were first identified as adenoviral contaminants in the 1960s^1-3^. In the nearly four decades following the earliest descriptions of recombinant AAV vectors^4,5^, hundreds of clinical trials have established that AAVs have the potential to be used safely for long-term expression of genetic payloads ^6-9^. There is, however, renewed concern about the safety of high-dose systemic AAV delivery following reports of adverse hepatotoxicity^10,11^ and several patient deaths^12,13^. The low therapeutic index of systemically-administered natural AAV serotypes necessitates high doses, particularly for the brain, highlighting the need for more efficient—and thus safer—AAVs. In recent years, the gene therapy field has focused on engineering novel capsids to address this problem and expand the window of therapeutic opportunity. In parallel, the neuroscience community has engineered several AAV variants that can traverse the restrictive blood-brain barrier (BBB). AAVs are now commonly used to systemically deliver genetically-encoded tools to the mouse brain^14-17^, such as GCaMP to detect intracellular calcium gradients^18^.

The engineering of neurotropic AAV variants in rodents has been catalyzed by advances in protein engineering, sequencing technologies, and understanding of AAV structure and function. For example, some of the first variants to efficiently traverse the BBB after intravenous (IV) administration in mice (AAV-PHP.B/eB) were engineered using Cre recombinase-based AAV targeted evolution (CREATE), which leverages Cre-transgenic mouse lines to impose additional selective pressure during library selections^14,15^. Implementing next-generation sequencing (NGS) and mutagenesis at different locations on the capsid surface has since led to variants with enhanced neurotropic properties, such as the ability to cross the BBB across different mouse strains, decreased transduction in non-CNS tissue, and biased tropism towards cell types in the brain^15-17,19-21^.

While AAV capsid engineering has enabled intravenous gene transfer to the rodent central nervous system (CNS), tools for non-human primates (NHPs) are sparse. Some capsids selected in rodents translate to the common marmoset^17^ (*Callithrix jacchus*), a New World primate species, but few translate to Old World primates, which are more closely evolutionarily related to humans and are well-established animal models of human cognition, neurodevelopment, neuroanatomy, and physiology^22-24^. Notably, despite its success in mice, the BBB-crossing tropism of AAV-PHP.B does not translate to the rhesus macaque (*Macaca mulatta*)^25,26^. In lieu of a vector for systemic gene transfer in macaques, researchers and clinicians resort to direct intraparenchymal injections to circumvent the BBB. However, due to limited spatial distribution, AAVs must typically be injected in multiple locations, invasively penetrating the brain parenchyma each time,^27-33^ with each surgery requiring resource-intensive pre-planning and real-time monitoring of infusions^28-31,34-39^. Recently, several groups have utilized intrathecal routes of administration via lumbar puncture (LP)^40^ or intra-cisterna magna (ICM)^41^ injection. However, these routes of administration have limited efficacy in the brain^41-45^, and some groups report adverse transduction in non-brain tissue, especially in the dorsal root ganglia^11,45-47^. To enable novel research in NHP animal models and for greater therapeutic translatability, it is imperative to advance AAV development for systemic gene transfer to the brains of Old World primates such as the macaque.

Here, we describe AAV.CAP-Mac, an engineered AAV9 variant that efficiently targets the CNS in both New World and Old World monkeys. CAP-Mac is biased towards neurons in infant Old World primates and vasculature in adult marmosets, making it the first vector described for systemic gene-delivery to vasculature in NHPs, and demonstrates significant improvement over AAV9 in adult rhesus macaque tissue (*ex vivo* and *in vivo*). CAP-Mac efficiently transduces neurons in the brains of at least two infant Old World primate species, the rhesus macaque and the green monkey (*Chlorocebus sabaeus*), achieving broader CNS distribution via IV than intrathecal administration^45,48^. Furthermore, CAP-Mac targets neuronal cells in the CNS more effectively than its parent AAV9. Demonstrating CAP-Mac’s immediate research utility, we capitalized on its neuronal bias to express (1) functional GCaMP for *ex vivo* two-photon (2P) calcium imaging and (2) a cocktail of fluorescent reporters for Brainbow-like^49,50^, multicolor labeling and morphological tracing in the rhesus macaque brain (Fig 1a). By characterizing CAP-Mac in multiple NHP species, we aim to both expand the AAV toolbox available to researchers interested in studying the Old World primate CNS and highlight the utility of engineering AAVs for increased translatability in higher order mammals.

## Results

### Using multiple non-human primate species to identify brain-enriched AAV variants

**Fig. 1:**
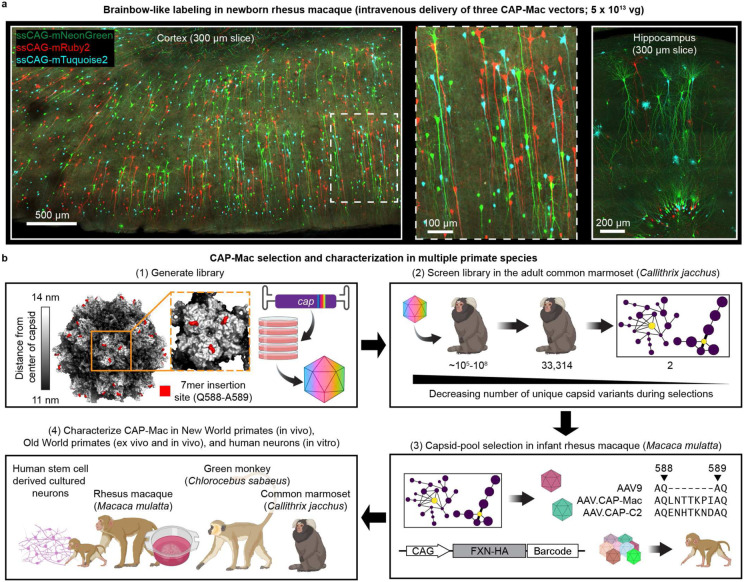
CAP-Mac selection and characterization strategy. **a**, AAV.CAP-Mac is a novel vector that enables brain-wide, systemic gene transfer in non-human primates. Representative images are shown from a newborn rhesus macaque brain expressing 3 fluorescent reporters delivered intravenously using AAV.CAP-Mac (5 x 10^13^ vg total dose, 4 weeks postinjection). **b,** Schematic of the CAP-Mac selection strategy. (1) CAP-Mac is an AAV9 variant that we selected from a library screened in the adult common marmoset. We generated diversity by introducing 21 NNK degenerate codons after Q588 in the AAV9 *cap* genome and produced the capsid library for *in vivo* selections in adult male marmosets. (2) In two rounds of selections, we intravenously administered 2 x 10^12^ vector genomes per marmoset, narrowing our variant pool with each round of selection. After the first round of selection, we recovered 33,314 unique amino acid sequences in the brain. For the second round of selection, we generated a synthetic oligo pool containing each unique variant plus a codon modified replicate (66,628 total sequences). After the second round of selection, we constructed network graphs of high-performing variants, and selected two capsids—AAV.CAP-Mac and AAV.CAP-C2—to be included in pool selections in newborn rhesus macaques. (3) For pool selections, we produced 8 capsids packaging ssCAG-hFXN-HA, each with a unique molecular barcode in the 3’ UTR. This construct design enabled us to assess protein expression of the pool by staining for the hemagglutinin (HA) epitope and quantify barcodes in viral DNA and whole RNA extracts. (4) We moved forward with individual characterization of AAV.CAP-Mac in various contexts (*ex vivo*, *in vitro*, *in vivo*) in multiple primate species.

Our overarching goal was to develop an AAV variant efficacious in NHPs after systemic administration. To do that, we used a multi-species screening and characterization strategy to select for variants with enhanced BBB-crossing tropism in NHPs (Fig. 1b). Briefly, we constructed a library as previously described by inserting 7mer sequences after Q588 in the structural *cap* gene of AAV9^14-16^ (Supplementary Fig. 1a-c). We initially screened this library in 2 rounds of selection in the adult marmoset (2 marmosets per round; 2 x 10^12^ vector genomes [vg] of viral library per marmoset via IV administration), where we identified 33,314 unique variants present in the brain.

In the past, we used our CREATE methodology to increase stringency during selections by only recovering variants that underwent cis-Cre-Lox mediated inversion^14,16^. However, since Cre-transgenic marmosets are not yet available, we pursued other strategies to compensate for the loss of this additional selective pressure. We previously demonstrated the utility of clustering capsid variants based on sequence similarity to generate network graphs as an aid in choosing variants for further characterization^16^. Briefly, we filtered variants based on user-defined performance criteria and clustered high-performing variants into network graphs (Supplementary Fig. 1d-g), wherein each node is a capsid variant, and each edge represents shared sequence identity between related variants (i.e., the pairwise reverse Hamming distance). We reasoned that this clustering analysis would let us efficiently sample variants from our selections while (1) limiting the number of animals used for individual characterization and (2) partially overcoming the absence of the CREATE selective pressure. Based on these network graphs, we chose two variants out of the 33,314 recovered from the marmoset for further characterization: AAV.CAP-Mac (CAP-Mac) and AAV.CAP-C2 (CAP-C2).

**Fig. 2:**
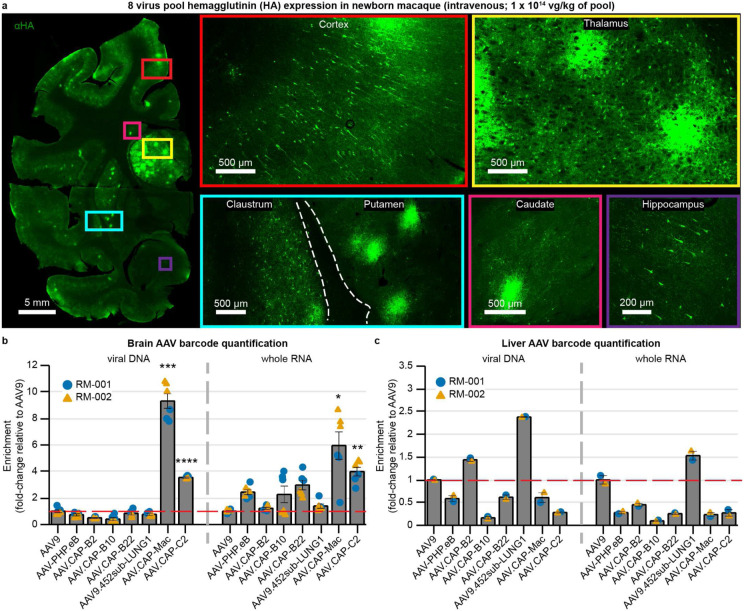
CAP-Mac outperforms other engineered variants in newborn rhesus macaque in pool testing. **a**, Representative images of expression in cortex, thalamus, caudate nucleus, putamen, hippocampus and claustrum after intravenous administration of 1 x 10^14^ vg/kg of an 8-capsid pool (1.25 x 10^13^ vg/kg of each variant) packaging hemagglutinin (HA) tagged human frataxin with a unique barcode in each capsid. **b**, **c**, Unique barcode enrichments in viral DNA (left) and whole RNA (right) extracts from the brain (**b**) and the liver (**c**) of two newborn rhesus macaques. Each data point represents the fold-change relative to AAV9 within each sample of tissue. Mean ± s.e.m. shown. The red dashed line denotes AAV9 performance in pool. One-way ANOVA using Tamhane’s T2 correction tested against AAV9 enrichment. (*P<0.05, **P<0.01, ***P<0.001, ****P<0.0001).

Following library selection in the adult marmoset, we used capsid-pool studies in newborn rhesus macaques to assess the translatability of several engineered AAVs to Old World primates. We pooled 8 capsid variants: AAV9, CAP-Mac, CAP-C2, and five other previously-engineered AAVs^15,17,51^. Each variant packaged a single-stranded human frataxin transgene fused to a hemagglutinin (HA) epitope tag under control of the ubiquitous CAG promoter (ssCAG-hFXN-HA) with a unique molecular barcode in the 3’ UTR. This construct design allowed us to assess protein expression of the virus pool via immunostaining of the HA epitope tag while also quantifying the relative enrichment of each unique barcode in DNA and RNA recovered from tissue. We administered 1 x 10^14^ vg/kg of the virus pool to 2 newborn rhesus macaques via the saphenous vein and, at 4 weeks post-injection, observed robust expression of the HA epitope throughout the brain (Fig. 2a). In the cortex and hippocampus, we observed single cells with clear projections that resemble the apical dendrites of pyramidal cells. Furthermore, we saw increased HA epitope expression in the thalamus and dorsal striatum (Fig. 2a, insets). When we quantified the relative enrichment of each barcode in the brain, we found that the CAP-Mac-delivered barcode was 9 and 6 times more abundant than the AAV9-delivered barcode in the viral DNA and total RNA, respectively (Fig. 2b). The CAP-C2-delivered barcode was approximately 4-fold enriched relative to the AAV9 barcode in both DNA and RNA extracts. Interestingly, the viral DNA levels of all other variants, which were originally selected in mice, were on par with AAV9. In the liver, CAP-Mac and CAP-C2 were negatively enriched, as were some of the previously-engineered controls known to be de-targeted from the liver in rodents^17^ (Fig. 2c).

### Characterization in newborn macaques and infant green monkeys: AAV.CAP-Mac efficiently transduces neurons in the CNS

**Fig. 3:**
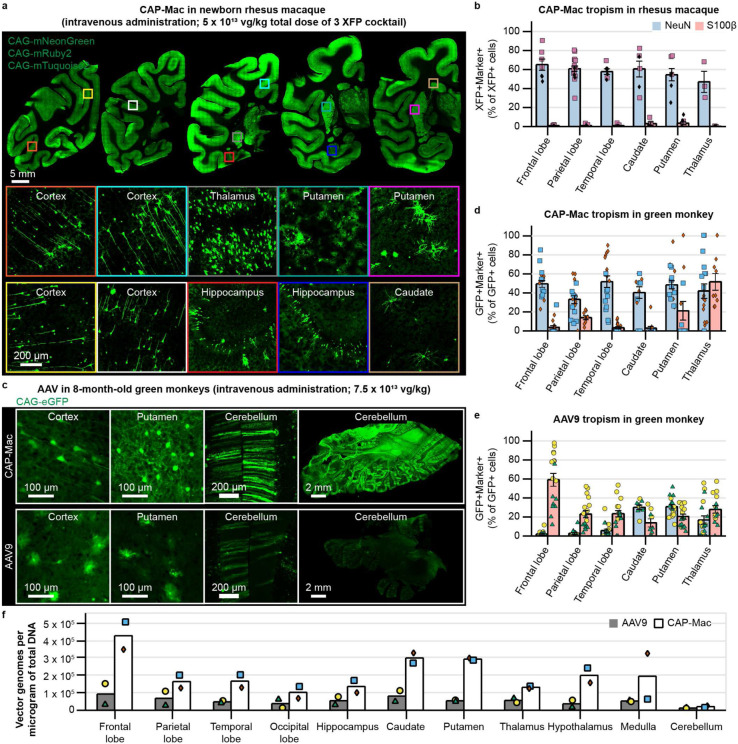
CAP-Mac is biased towards neurons throughout infant green monkey and newborn rhesus macaque brains. **a**, Distribution of CAP-Mac expression in 2-day-old rhesus macaques (5 x 10^13^ vg/kg via intravenous administration) across coronal slices showing fluorescent reporter expression (ssCAG-mNeonGreen, ssCAG-mRuby2, ssCAG-mTurquoise2) in cortical and subcortical brain regions (insets). Imaging channels of reporters are identically pseudo-colored. **b**, Colocalization of fluorescent reporters with NeuN (neurons) or S100β (astrocytes) in 2-day-old rhesus macaques treated with CAP-Mac. Values are reported as a percentage of all XFP+ cells. **c,** Representative images from 8-month-old green monkeys dosed with CAP-Mac (top) or AAV9 (bottom) packaging ssCAG-eGFP (7.5 x 10^13^ vg/kg via intravenous administration). **d**, **e**, Colocalization of fluorescent reporters with NeuN (neurons) or S100β (astrocytes) in infant green monkeys treated with CAP-Mac (**d**) or AAV9 (**e**). Values are reported as a percentage of all GFP+ cells. **f**, Distribution of CAP-Mac and AAV9-delivered eGFP transgene in 11 brain regions of green monkeys. Each data point represents measured vector genomes per microgram of total DNA in a section of tissue from each region and monkey. Mean ± s.e.m. shown.

Because CAP-Mac outperformed AAV9 and other engineered variants in our pool study, we moved forward with single characterization in two species of Old World primates. In the newborn rhesus macaque, we intravenously administered a cocktail of CAP-Mac vectors (5 x 10^13^ vg/kg total dose via the saphenous vein) packaging 3 different fluorescent reporters under control of the CAG promoter. Fluorescent protein (XFP) expression was observed in multiple coronal slices along the anterior-posterior axis (Fig. 3a) and was robust in all four lobes of cortex and in subcortical areas like the dorsal striatum and hippocampus. While expression was particularly strong in several nuclei of the thalamus (e.g., lateral and medial nuclei, lateral geniculate nucleus, pulvinar nucleus), we noted that expression was not found in all brain regions (e.g. the amygdala). Even with a ubiquitous promoter, we observed expression primarily in NeuN+ neurons (mean [XFP+NeuN+]/XFP+ between 47-60% across sampled brain regions) and rarely in S100β+ astrocytes (mean [XFP+S100β+]/XFP+ between 0-3%; Fig. 3b). We also attempted to deliver CAP-Mac via LP administration in newborn rhesus macaques, but found that efficiency throughout the brain was noticeably decreased compared to IV administration (Supplementary Fig. 2). Expression was especially low in subcortical structures, as reported previously^41-45^.

AAV variants engineered for BBB-crossing in mice are known to have strain-dependent behavior^16,26,52-54^. Therefore, in parallel with the rhesus macaque experiments, we characterized CAP-Mac in green monkeys, another Old World primate species. We administered either AAV9 or CAP-Mac packaging green fluorescent protein under control of CAG (ssCAG-eGFP) to individual 8-month-old monkeys (7.5 x 10^13^ vg/kg via the saphenous vein). In the CAP-Mac-dosed green monkeys, we saw broad and strong expression in cortex and various subcortical regions, including the putamen (Fig. 3c), consistent with the capsid-pool (Fig. 2a) and rhesus macaque (Fig. 3a and b) results. We saw particularly strong eGFP expression throughout the cerebellum in the CAP-Mac-dosed green monkey. Except in the thalamus, CAP-Mac eGFP expression was again found primarily in neurons (mean [GFP+NeuN+]/GFP+ between 33-51%) and not astrocytes (mean [GFP+ S100β+]/GFP+ between 3-21%; Fig. 3d). In the thalamus, 42% of GFP+ cells were neurons and 51% astrocytes. In AAV9-dosed monkeys, AAV9 eGFP expression was primarily biased towards astrocytes in cortex (mean [GFP+S100β+]/GFP+ between 23-59%) with low neuronal transduction (mean [GFP+NeuN+]/GFP+ between 2-10%; Fig. 3e), which is consistent with other reports^41,45,55,56^. Notably, recovered CAP-Mac transgenes were more abundant throughout the brain compared to AAV9, suggesting overall higher brain penetrance of CAP-Mac (Fig. 3f and Supplementary Fig. 3a). Interestingly, the cerebellum contained the fewest vector genomes per microgram of DNA in both CAP-Mac monkeys despite strong eGFP expression, most likely due to the high density of cells and processes within the cerebellum^57,58^. In most non-brain tissue, eGFP biodistribution and expression was comparable between CAP-Mac- and AAV9-treated animals (Supplementary Fig. 3). It should be noted that the cell-type tropism differences between CAP-Mac and AAV9 in the brain may apply to non-brain tissue as well, with each vector transducing distinct cell types. Even in highly homogenous cell populations, there is significant viral infection variability^59-61^, so measuring AAV genomes in bulk may not fully reflect capsid penetrance in tissue across variants and cell types.

### Experimental utility of CAP-Mac to study the macaque brain

**Fig. 4:**
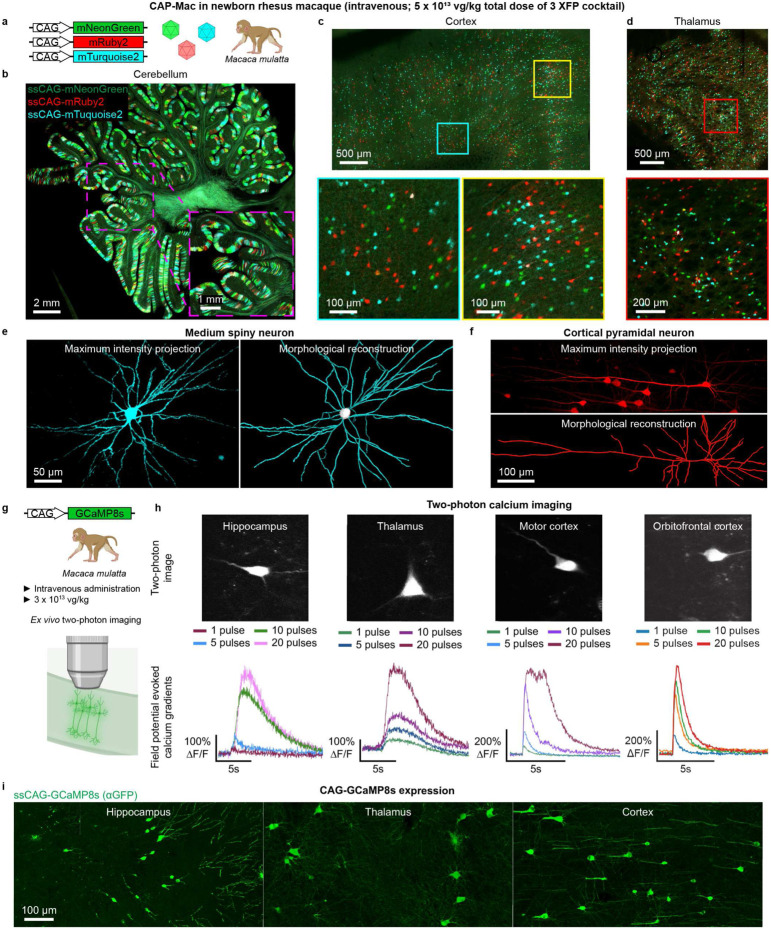
Experimental utility of CAP-Mac for interrogation of the newborn rhesus macaque brain. **a**-**f**, CAP-Mac packaging three fluorescent reporters (**a**) to generate Brainbow-like labeling in rhesus macaque cerebellum (**b**), cortex (**c**), and thalamus (lateral geniculate nucleus) (**d**), enabling morphological reconstruction of neurons (**e** and **f**). **g-i,** Non-invasively delivering ssCAG-GCaMP8 using CAP-Mac (**g**) for *ex vivo* two-photon imaging (**h**) and brain-wide GCaMP expression (**i**).

The NIH BRAIN initiative emphasizes the priority of developing novel tools for genetic modulation in NHPs to inform further understanding of the human brain^62^. Accordingly, we explored if we could leverage CAP-Mac’s neuronal tropism in newborn macaques to deliver genetically-encoded reporters to interrogate the brain. First, we tested whether CAP-Mac can be used as a non-invasive method to define neuronal morphology. Having administered a cocktail of 3 CAP-Mac vectors packaging different fluorescent proteins (Fig. 4a), we attempted Brainbow-like labeling^15,49,50^ in an Old World primate. We observed widespread expression of all 3 fluorescent proteins in cerebellum, cortex, and the lateral geniculate nucleus of the thalamus (Fig. 4b-d). In the cerebellum and thalamus, we observed a high density of transduced cells, and the highest proportion of co-localization of 2 or 3 fluorescent proteins. However, co-localization of multiple fluorescent proteins was rare, suggesting that co-infection was uncommon after systemic administration. With broad and robust expression of fluorescent proteins throughout the brain, we were able to assemble morphological reconstructions of medium spiny neurons (Fig. 4e) and cortical pyramidal cells (Fig. 4f).

In a second set of experiments, we sought to use CAP-Mac to express functional GCaMP throughout the CNS of infant macaques (Fig. 4g). Given the experimental complexity and limited accessibility of NHPs, when designing our GCaMP experiments, we performed initial cargo screening in mice. We therefore first characterized CAP-Mac in three mouse strains. We found that the neuronal bias of CAP-Mac extended to mice when delivered to the adult brain through ICV (Supplementary Fig. 5a) but not IV administration, where it primarily transduced cells with vasculature morphology (Supplementary Fig. 5b), with no apparent differences between the three mouse strains. We also found that in P0 C57BL/6J mice, IV-administered CAP-Mac was expressed in various cell types in the brain, including neurons, astrocytes, and vasculature (Supplementary Fig. 5c). Given the strong neuronal tropism of CAP-Mac following ICV administration, we used this method to screen two genetic cargos (either one-component or two-component vectors) in mice prior to applying them to NHPs (Supplementary Fig. 5d-g). Given our results from this cargo selection in mice, we moved forward with a one-component system using the CAG promoter.

We intravenously delivered ssCAG-GCaMP8s to newborn macaques (3 x 10^13^ vg/kg via the saphenous vein) and after 4-6 weeks of expression, we removed tissue for *ex vivo* 2P imaging. In the hippocampus, thalamus, and cortex we successfully recorded field potential-evoked calcium gradients in GCaMP-expressing cells (Fig. 4h). Cells were responsive to restimulation throughout the experiment and, importantly, the mean peak ΔF/F of GCaMP signal increased with increases in number of field potential pulses (Supplementary Fig. 4a). Cellular calcium dynamics differed across the four sampled brain regions (Supplementary Fig. 4b-e). Consistent with our previous profiling, we saw GCaMP expression primarily in cell types with neuronal morphology throughout the brain (Fig. 4i).

### Human cultured neurons: AAV.CAP-Mac strongly transduces human neurons compared to AAV9

**Fig. 5:**
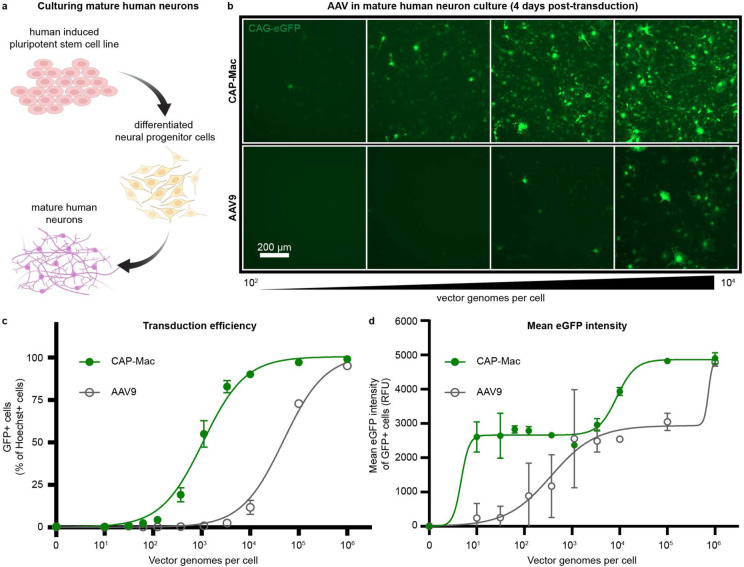
CAP-Mac is more potent at transducing human cultured neurons compared to AAV9. **a**, Differentiation process starting with a human induced pluripotent stem cell line that was differentiated into neural progenitor cells, which were further differentiated into mature neurons. **b**, Representative images of cultured human neurons after 4 days of incubation with either CAP-Mac (top) or AAV9 (bottom) packaging CAG-eGFP across 4 doses of AAV, ranging from 10^2^-10^4^ vector genomes per cell. **c**, **d**, Dose response curves of AAV9 and CAP-Mac in mature human neuron culture measuring transduction efficiency (**c**) and mean eGFP intensity (**d**).

Given the efficacy of CAP-Mac in penetrating the brain of infant Old World primates and motivated by our observation that CAP-Mac primarily transduces neurons, we wanted to test whether CAP-Mac offered any improvement over its parent capsid, AAV9, in transducing human neurons. We differentiated cultured human-derived induced pluripotent stem cells (iPSCs) into mature neurons (Fig. 5a) and incubated them with CAP-Mac or AAV9 packaging ssCAG-eGFP at doses ranging from 0 vg/cell to 10^6^ vg/cell. We found that eGFP expression was noticeably increased in CAP-Mac-administered cultures compared to AAV9-administered cultures (Fig. 5b). AAV9 transduction achieved an efficiency of EC_50_=10^4.68^ vg/cell, while CAP-Mac achieved EC_50_=10^3.03^ vg/cell (Fig. 5c), a 45-fold increase in potency (P=0.0023 using two-tailed Welch’s t-test). Average per-cell eGFP expression measured across transduced cells fit a biphasic step function, with CAP-Mac reaching the first plateau at a dose roughly two orders of magnitude lower than AAV9 (Fig. 5d). Overall, the increased potency of CAP-Mac in transducing mature human neurons *in vitro* is consistent with the neuronal tropism we observed in infant Old World primates, suggesting a similar mechanism of neuronal transduction across species.

### Adult non-human primate tissue: an improved vector compared to AAV9

**Fig. 6:**
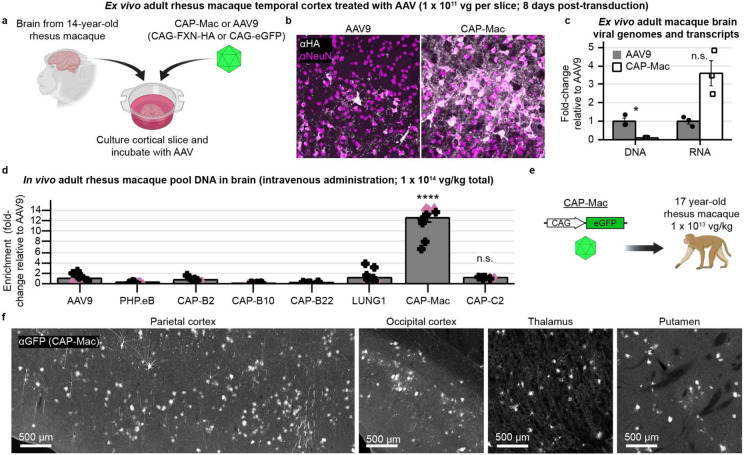
Characterization in adult rhesus macaque. **a,** AAV in cortical slice *ex vivo* taken from a 14-year-old rhesus macaque. **b**, CAP-Mac is more efficient at transducing neurons in gray matter of cortex. **c**, Quantification demonstrates that CAP-Mac-delivered transgene is better at producing RNA but not DNA compared to AAV9-delivered transgene. Two-tailed Welch’s t-test (*P<0.05). **d-f**, AAV in adult rhesus macaques *in vivo*. **d**, Recovered DNA from adult macaque administered with 8-capsid pool. One-way ANOVA using Tamhane’s T2 correction tested against AAV9 enrichment. (****P<0.0001). **e**, We injected 1 x 10^13^ vg of CAP-Mac packaging a CAG-eGFP into one 17-year-old rhesus macaque to assess CAP-Mac protein expression. **g**, CAP-Mac-mediated eGFP expression visualized after amplification with GFP antibody. Mean ± s.e.m. shown.

Infant NHPs offer several logistical advantages for AAV characterization. For instance, they are more likely to be seronegative for neutralizing AAV antibodies, and their smaller body weight requires less vector to be produced for a given dose. While the mammalian BBB is fully formed by birth—including intact tight junctions which give rise to the BBB’s unique functionality to limit passive molecular transport into the brain—dynamic molecular and cellular processes occurring during development may make the BBB more permissive^63-66^. We therefore wanted to characterize CAP-Mac’s tropism in adult macaque to determine tropism differences across developmental stages. To further de-risk our characterization, we first chose to test CAP-Mac in adult rhesus macaque slices *ex vivo* (Fig. 6a). In the gray matter of cultured cortical slices, cargo delivered by CAP-Mac, but not AAV9, co-localized with NeuN+ cells, consistent with our previous results (Fig. 6b). Unexpectedly, only 9% as many CAP-Mac viral genomes were recovered as AAV9 genomes, but 3.6-fold more viral transcripts were recovered from CAP-Mac-treated slices than from AAV9-treated slices (Fig. 6c).

While informative, *ex vivo* characterization does not assess BBB penetration, so we next tested CAP-Mac in adult macaques *in vivo.* We injected two adult rhesus macaques with the same AAV pool that we used in infants and found that CAP-Mac-delivered genomes were 13-fold more abundant in the brain than AAV9 (Fig. 6d). Again, the variants originally selected in mice were all less efficient than AAV9, but CAP-C2 was 1.2-fold more efficient than AAV9. To further assess protein expression, we injected CAP-Mac packaging CAG-eGFP (1 x 10^13^ vg/kg total dose via the saphenous vein) into a 17-year-old adult rhesus macaque (Fig. 6e). At the protein level, we observed CAP-Mac-delivered eGFP expression (visualized via eGFP antibody amplification) in parts of the cortex and thalamus, while eGFP expression was absent in other regions of the brain.

Finally, since CAP-Mac was originally identified using *in vivo* selections in the adult common marmoset, we also wanted to characterize the vector in the selection species. As in the adult macaque experiment, we injected CAP-Mac and AAV9 into adult marmosets (3.8 and 5.8 years old). To our surprise, we found that the tropism of CAP-Mac in adult marmoset was biased primarily towards the GLUT1+ vasculature (Supplementary Fig. 6), consistent with our results in adult mice.

## Discussion

Here we describe AAV.CAP-Mac, an engineered AAV9 variant with increased efficiency for brain-wide transgene expression in multiple NHP species.

By comprehensively characterizing CAP-Mac in multiple rodent strains and NHP species, across ages and administration routes, we found that CAP-Mac tropism varies depending on species, developmental state, and route of administration (Supplementary Table 5). This is not surprising given the heterogeneity of the BBB across species and populations^67-69^, a challenge noted in other AAV engineering efforts^70-72^. Performing such a comprehensive characterization of CAP-Mac in multiple contexts was beneficial for two reasons. First, we discovered that CAP-Mac was primarily biased towards the brain endothelium in the adult marmoset, to our knowledge the first description of a systemic vector that targets the vasculature in adult marmosets. Second, by testing alternative routes of administration in mice, we found that CAP-Mac tropism is shifted towards neurons after ICV administration, mirroring the tropism in newborn macaques and giving us a method to assess expression and functional activity of GCaMP configurations before applying them to NHPs (Supplementary Fig. 5). In lieu of a cross-species capsid with conserved tropism and efficiency in rodents and NHPs, this approach can be a valuable tool for users to validate capsid-cargo combinations in mice prior to use in NHPs.

*In vivo* AAV capsid selections have been primarily conducted in mice, in part due to the utility of Cre-transgenic mouse lines to increase selective pressure, which can yield neurotropic capsids in as few as two rounds of selection^14,16,17^. However, these engineered variants have largely failed to translate to NHPs^25,26^. The notable exceptions are AAV.CAP-B10 and AAV.CAP-B22, which were identified using multiplexed-CREATE (M-CREATE)^16^ selections in mice and retain their BBB crossing and reduced liver tropism in the common marmoset^17^, a New World primate. However, our pool testing here showed that these variants perform only on par with AAV9 in delivering DNA to the brains of infant macaques, an Old World primate. While mice last shared a common ancestor with humans approximately 80-90 million years ago (mya), marmosets and macaques are believed to have shared their last ancestors with humans 35-40 mya and 25-30 mya, respectively^73^. Given this evolutionary distance, it is not surprising that most variants selected in mice have failed to translate to Old World primates, and vice versa. Interestingly, our pool studies in macaques showed that variants identified via Cre-independent selections in marmosets and chosen using network graphs (CAP-Mac and CAP-C2) generally outperformed variants identified via Cre-dependent selections in mice (Fig. 2b). This suggests that while enhancing selective pressure is important when evolving engineered AAVs *in vivo,* it is also vital to consider the evolutionary relatedness between the selection and target species. Notably, several transgenic marmoset lines are currently available^74,75^, and the generation of Cre-transgenic marmosets is underway^76^, offering the potential to perform M-CREATE in NHPs. Given that the evolutionary distance between mice and marmosets (40-55 mya) is slightly larger than that between marmosets and humans (35-40 mya), the observation that AAV.CAP-B10 and AAV.CAP-B22 retain their BBB-crossing tropisms in marmosets offers hope that NHP selections can identify capsid variants efficacious in humans.

The overarching goal of this study was to define and disseminate a suite of genetic tools to study the NHP brain, especially in Old World primates. This includes characterizing cargo that can be delivered by CAP-Mac, as both self and non-self proteins (e.g. GFP) are known to be immunogenic in certain contexts^77-80^. To that end, we describe two functional cargos for studying the Old World primate brain: (1) a cocktail of three fluorescent reporters for Brainbow-like^49,50^ labeling, and (2) GCaMP8s for optical interrogation of *ex vivo* neuronal activity. Encouragingly, our GCaMP recordings demonstrate that cells expressing CAP-Mac-delivered molecular sensors are physiologically active and healthy in *ex vivo* rhesus macaque slices. To our knowledge, this is the first description of using a non-invasive, systemic vector to deliver genetically-encoded sensors to the macaque brain, a transformational technique previously limited to rodents. Notably, none of the rhesus macaques dosed in this study experienced adverse events or abnormal liver function and assessment by an independent pathologist confirmed that the vectors were administered safely (Supplementary Fig. 7 and Supplementary Table 6). Moving forward, we expect CAP-Mac-mediated gene transfer to help illuminate circuit connectivity and neuronal function in the macaque brain^81,82^ and, more generally, assist major efforts such as the NIH BRAIN Initiative^62^ to understand the inner workings of the primate CNS.

In addition to CAP-Mac’s utility as a tool to study the primate brain, it is also a compelling potential delivery vehicle for genetic medicine in humans. It provides an unprecedented opportunity to deepen our understanding of pharmacodynamics in Old World primate models^30,83,84^ and its broad and uniform distribution throughout the CNS opens access to subcortical and midbrain regions for neuroscience researchers, currently difficult in NHPs^41-45^. Additionally, CAP-Mac’s enhanced transduction of cultured human neurons supports its potential as a gene-delivery vehicle in humans. Overall, the success of the capsid engineering approach we describe here offers a roadmap for developing the next class of translational gene therapies with improved safety and efficacy profiles.

## Methods

### AAV DNA library generation

We initially generated diversity at the DNA level, which we then used to produce transfection material to produce the AAV capsid library. For the round 1 library, we introduced this genetic diversity using primers containing degenerate nucleotides inserted between amino acids (AA) 588 and 589^14–16^ (VP1 numbering; Supplementary Fig. 1a). We used a reverse primer containing 21 degenerate nucleotides ([NNK] x 7) to randomly generate PCR fragments containing unique 7mer sequences inserted into the *cap* gene. For the round 2 DNA library, we used a synthetic oligo pool (Twist Bioscience) as a reverse primer, encoding only variants selected for further screening (66,628 DNA oligos total: 33,314 variants recovered after round 1 selections plus a codon-modified replicate of each). All reverse primers contained a 20 bp 5’ overhang complementary to the *cap* sequence near the AgeI restriction enzyme sequence and were paired with a forward primer containing a 20 bp 5’ overhang near the XbaI restriction enzyme sequence. We then inserted the PCR fragments containing the diversified region into the rAAV-ΔCAP-in-cis-Lox plasmid via Gibson assembly to generate the resulting AAV DNA library, rAAV-CAP-in-cis-Lox, using NEBuilder HiFi DNA Assembly Master Mix (New England Biolabs, E2621).

### AAV capsid library production

We generated AAV capsid libraries according to previously published protocols^16,85^. Briefly, we transfected HEK293T cells (ATCC, CRL-3216) in 150 mm tissue culture plates using transfection grade, linear polyethylenimine (PEI; Polysciences, Inc). In each plate, we transfected 4 plasmids: (1) the assembled rAAV-Cap-in-cis-Lox AAV DNA library, which is flanked by inverted terminal repeats (ITR) required for AAV encapsidation; (2) AAV2/9 REP-AAP-ΔCAP, which encodes the REP and AAP supplemental proteins required for AAV production with the C-terminus of the *cap* gene excised to prevent recombination with the AAV DNA library and subsequent production of replication-competent AAV; (3) pHelper, which encodes the necessary adenoviral proteins required for AAV production; and (4) pUC18, which contains no mammalian expression vector but is used as filler DNA to achieve the appropriate nitrogen-to-phosphate ratio for optimal PEI transfection. During preparation of the PEI-DNA mixture, we added 10 ng of our AAV DNA library (rAAV-Cap-in-cis-Lox) for every 150 mm dish and combined AAV2/9 REP-AAP-ΔCAP, pUC18, and pHelper in a 1:1:2 ratio, respectively (40 μg of total DNA per 150 mm dish). At 60 hours post-transfection, we purified AAV capsid library from both the cell pellet and media using polyethylene glycol precipitation and iodixanol gradient ultracentrifugation. Using quantitative PCR, we then determined the titer of the AAV capsid libraries by amplifying DNaseI-resistant viral genomes relative to a linearized genome standard according to established protocols^85^.

### Marmoset experiments

#### Capsid library selections

All marmoset (*Callithrix jacchus*) procedures were performed at the National Institutes of Mental Health (NIMH) and approved by the local Institutional Animal Care and Use Committee (IACUC). Marmosets were born and raised in NIMH colonies and housed in family groups under standard conditions of 27°C and 50% humidity. They were fed ad libitum and received enrichment as part of the primate enrichment program for NHPs at the National Institutes of Health. For all marmosets used in this study, there were no detectible neutralizing antibodies at a 1:5 serum dilution prior to IV infusions (conducted by The Penn Vector Core, University of Pennsylvania). They were then housed individually for several days and acclimated to a new room before injections. Four adult males were used for the library screening, 2 each for first- and second-round libraries. The day before infusion, the animals’ food was removed. Animals were anesthetized with isoflurane in oxygen, the skin over the femoral vein was shaved and sanitized with an isopropanol scrub, and 2 x 10^12^ vg of the AAV capsid library was infused over several minutes. Anesthesia was withdrawn and the animals were monitored until they became active, upon which they were returned to their cages. Activity and behavior were closely monitored over the next 3 days, with daily observations thereafter.

At 4 weeks post-injection, marmosets were euthanized (Euthanasia, VetOne) and perfused with 1X phosphate-buffered saline (PBS). After the round 1 library, the brain was cut into 4 coronal blocks, flash frozen in 2-methylbutane (Sigma Aldrich, M32631), chilled with dry ice, and stored at −80°C for long term storage. After the round 2 library, the brain was cut into 6 coronal blocks and, along with sections of the spinal cord and liver, was flash frozen and stored at −80°C for long term storage.

#### Individual characterization of AAV in marmosets

Two adult common marmosets (*Callithrix jacchus*) were used for this experiment: Conan (male, 2.8 years old, 0.386 kg) and Sandy (female, 5.8 years old, 0.468 kg; see Supplementary Table 4 for more details). They were housed under standard conditions of 27°C and 50% humidity, with ad libitum access to food and water. All animals were group housed, and experiments were performed in the Cortical Systems and Behavior Laboratory at University of California San Diego (UCSD). All experiments were approved by the UCSD Institutional Animal Care and Use Committee (IACUC). The day before infusion, the animals’ food was removed.

Animals were anesthetized with ketamine (Ketaset, Zoetis 043-304, 20mg/kg), the skin over the saphenous vein was shaved and sanitized with an isopropanol scrub, and 2 x 10^13^ vg/kg of AAV was infused over 5 minutes. The animals were monitored until they became active, upon which they were returned to their cages. Activity and behavior were closely monitored over the next 3 days, with daily observations thereafter. Blood samples were taken at days 1, 7, 14, 21 and 31 to measure viral concentration in plasma.

At 31 days post-injection, marmosets were anesthetized with ketamine as described earlier and then euthanized (Euthasol, Virbac 200-071, 1mL/kg) and perfused with 1X phosphate-buffered saline (PBS). Brains and organs were cut in half, and one half was flash-frozen in 2-methylbutane (Sigma Aldrich, M32631), chilled with dry ice, and stored at −80°C. The other half was fixed in 4% PFA (Thermo Scientific, J19943-K2) overnight and then stored at 4°C in PBS-Azide (Sigma Aldrich, S2002-100G, 0.025%). Samples were then shipped to California Institute of Technology (Caltech) for analysis.

### Viral library DNA extraction and NGS sample preparation

We previously reported that viral library DNA and endogenous host RNA can be isolated using TRIzol by precipitating nucleic acid from the aqueous phase^14,16^. Therefore, to extract viral library DNA from marmoset tissue, we homogenized 100 mg of spinal cord, liver, and each coronal block of brain in TRIzol (Life Technologies, 15596) using a BeadBug (Benchmark Scientific, D1036) and isolated nucleic acids from the aqueous phase according to the manufacturer’s recommended protocol. We treated the reconstituted precipitate with RNase (Invitrogen, AM2288) and digested with SmaI to improve downstream viral DNA recovery via PCR. After digestion, we purified with a Zymo DNA Clean and Concentrator kit (D4033) according to the manufacturer’s recommended protocol and stored the purified viral DNA at −20°C.

To append Illumina adapters flanking the diversified region, we first PCR-amplified the region containing our 7mer insertion using 50% of the total extracted viral DNA as a template (25 cycles). After Zymo DNA purification, we diluted samples 1:100 and further amplified around the variable region with 10 cycles of PCR, appending binding regions for the next PCR reaction. Finally, we appended Illumina flow cell adapters and unique indices using NEBNext Dual Index Primers (New England Biolabs, E7600) via 10 more cycles of PCR. We then gel-purified the final PCR products using a 2% low-melting point agarose gel (ThermoFisher Scientific, 16520050) and recovered the 210 bp band.

For the second-round library only, we also isolated the encapsidated AAV library ssDNA for NGS to calculate library enrichment scores, a quantitative metric that we used to normalize for differences in titer of the various variants in our library (see ref. 16 and the next section). To isolate the encapsidated viral genomes, we treated the AAV capsid library with DNaseI and digested capsids using proteinase K. We then purified the ssDNA using phenol-chloroform, amplified viral transgenes by 2 PCR amplification steps to add adapters and indices for Illumina NGS, and purified using gel electrophoresis. This viral library DNA, along with the viral DNA extracted from tissue, was sent for deep sequencing using an Illumina HiSeq 2500 system (Millard and Muriel Jacobs Genetics and Genomics Laboratory, Caltech).

### NGS read alignment, analysis, and generation of network graphs

Raw fastq files from NGS runs were processed with custom-built scripts (https://github.com/GradinaruLab/protfarm and https://github.com/GradinaruLab/mCREATE)^16^. For the first-round library, the pipeline to process these datasets involved filtering to remove low-quality reads, utilizing a quality score for each sequence, and eliminating bias from PCR-induced mutations or high GC-content. The filtered dataset was then aligned by a perfect string match algorithm and trimmed to improve the alignment quality. We then displayed absolute read counts for each variant during the sequencing run within each tissue, and all 33,314 variants that were found in the brain were chosen for round 2 selections.

After round two selections, we performed the same analysis to display variant absolute read count of the injected virus library and of each variant within each tissue. Additionally, we calculated the library enrichment^16^ for each variant within each tissue:

$${\hat{RC}}_{x,injected\;library}=\frac{RC_{x,injected\;library}}{\sum_{i=1}^{N_{injected\;library}}RC_{i,injected\;library}}\;\;{\hat{RC}}_{x,tissue}=\frac{RC_{x,virus}}{\sum_{i=1}^{N_{tissue}}RC_{i,tissue}}\;\;library\;enrichment=log_{10}(\frac{{\hat{RC}}_{x,injected\;library}}{{\hat{RC}}_{x,tissue}})$$

such that for a given sample *y* (e.g. the injected virus library or a tissue sample), *RC*_*x,y*_ is the absolute read count of variant *x, N*_*y*_ is the total number of variants recovered, and ${\hat{RC}}_{x,y}$ is the normalized read count.

To construct the CAP-Mac sequence clustering graph, we filtered the round 2 NGS data based on the following criteria: (1) ≥ 100 read count in the injected library sample (24,186/33,314 variants), (2) ≥ 0.7 library enrichment score in more than 2 brain samples (415 variants), and (3) at least 2 more brain samples with ≥ 0.7 library enrichment than brain samples with < −0.7 library enrichment (323 variants). To construct the CAP-C2 sequence graph, we filtered the round 2 NGS data based on the following criteria: (1) ≥ 100 read count in the injected library sample and (2) both codon replicates present in at least 2 brain samples with ≥ 0.7 library enrichment (95 variants). These variants were then independently processed to determine pair-wise reverse Hamming distances (https://github.com/GradinaruLab/mCREATE) and clustered using Cytoscape (ver. 3.9.0) as described previously^16^. Networks presented show capsid variants (nodes) connected by edges if the pair-wise reverse Hamming distance is ≥ 3.

### Cloning individual AAV capsid variants

For single variant characterization, we cloned new variant plasmids by digesting a modified version of the pUCmini-iCAP-PHP.eB (Addgene ID: 103005) backbone using MscI and AgeI. We designed a 100 bp primer that contained the desired 21 bp insertion for each capsid variant and the regions complementary to the AAV9 template with ~20 bp overlapping the digested backbone. We then assembled the variant plasmid using NEBuilder HiFi DNA Assembly Master Mix, combining 5 μL of 200 nM primer with 30 ng of digested backbone in the reaction mixture.

### Individual AAV production and purification

To produce variants for pool testing, we followed our previously published protocol^85^ using 150 mm tissue culture dishes. For individual AAV.CAP-Mac and AAV9 characterization *in vivo* and *in vitro,* we adopted our published protocol to utilize ten-layer CellSTACKs (Corning, 3320) to efficiently produce viruses at high titer to dose rhesus macaques and green monkeys. Specifically, we passaged 20 150-mm dishes at approximately 70% confluency into a 10-layer CellSTACK 24 h before transfection. On the day of transfection, we prepared the DNA-PEI transfection mixture for 40 150-mm dishes and combined the transfection mixture with media and performed a complete media change for the CellSTACK. We collected and changed media at 72 h post-transfection similarly to production in 150 mm dishes. At 120 h post-transfection, we added ethylenediaminetetraacetic acid (EDTA, Invitrogen, 15575020) to a final concentration of 10 mM and incubated at 37°C for 20 min, occasionally swirling and tapping the sides of the CellSTACK to detach the cells. We then removed the media and cell mixture and proceeded with the AAV purification protocol^85^. Of note, during the buffer exchange step after ultracentifugation, we used centrifugal protein concentrators with polyethersulfone membranes (Thermo Scientific, 88533) instead of Amicon filtration devices and used Dulbecco’s PBS supplemented with 0.001% Pluronic^®^ F-68 (Gibco, 24040032).

### Rodent experiments

All rodent procedures were performed at Caltech and were approved by the local IACUC. We purchased C57BL/6J (000664), BALB/cJ (000651), and DBA/2J (000671) mice (all males, 6–8 weeks old) from The Jackson Laboratory. For IV administration in mice, we delivered 5 x 10^11^ vg of virus through the retro-orbital sinus^85,86^ using a 31 G insulin syringe (BD, 328438). For intracerebroventricular administration in mice, we injected 5 x 10^10^ or 1.5 x 10^11^ vg into the lateral ventricle. Briefly, we anesthetized mice using isoflurane (5% for induction, 1-3% for maintenance) with 95% O_2_/5% CO_2_ (1 L/min) and mice were head-fixed in a stereotaxic frame. After shaving the head and sterilizing the area with chlorohexidine, we administered 0.05 mL of 2.5 mg/mL bupivacaine subcutaneously, and a midline incision was made and the skull was cleaned of blood and connective tissue. After leveling the head, burr holes were drilled above the lateral ventricles bilaterally (0.6 mm posterior to bregma, 1.15 mm from the midline). Viral vectors were aspirated into 10 μL NanoFil syringes (World Precision Instruments) using a 33-guage microinjection needle, and the needle was slowly lowered into the lateral ventricle (1.6 mm from the pial surface). The needle was allowed to sit in place for approximately 5 min and 3-5 μL of viral vector was injected using a microsyringe pump (World Precision Instruments, UMP3) and pump controller (World Precision Instruments, Mircro3) at a rate of 300 nL/min. All mice received 1 mg/kg of buprenorphine SR and 5 mg/kg of ketoprofen subcutaneously intraoperatively and 30 mg/kg of ibuprofen and 60 mg/kg of Trimethoprim/Sulfamethoxazole (TMPS) for 5 days post-surgery. After 3 weeks of expression, all mice were perfused with PBS and fixed in 4% paraformaldehyde (PFA). All organs were extracted, incubated in 4% PFA overnight, transferred into PBS supplemented with 0.01% sodium azide, and stored at 4°C for long-term storage. We sliced the brain into 100 μm sections by vibratome (Leica Biosystems, VT1200S), mounted in Prolong Diamond Antifade (Invitrogen, P36970), and imaged using a confocal microscope (Zeiss, LSM 880).

### Rhesus macaque experiments

All rhesus macaque (*Macaca mulatta)* procedures were performed at the California National Primate Research Center (CNPRC) at UC Davis and were approved by the local IACUC. Neonate macaques (0.45-1.4 kg) were weaned at birth. Within the first month, macaques were infused with AAV vectors either intravenously (IV) or intrathecally (LP). For IV injections, animals were anesthetized with ketamine (0.1 mL) and the skin over the saphenous vein was shaved and sanitized. AAV (between 2 x 10^13^ and 1 x 10^14^ vg/kg) was slowly infused into the saphenous vein over ~1 min in < 0.75 mL of phosphate buffered saline. For LP injections, animals were administered a sedative intramuscularly and the area of skin at the neck was shaved and aseptically prepared. A needle was advanced into the cisterna magna to remove a small amount of CSF proportional to the amount of fluid injected. Then, a sterile syringe containing the sterile preparation of the AAV (1.5 x 10^12^ or 2.5 x 10^13^ vg/kg) proportional to the amount of fluid collected was aseptically attached and slowly injected. All animals were monitored during recovery from sedation, throughout the day, and then daily for any adverse findings. All monkeys were individually housed within sight and sound of conspecifics. Tissue was collected 4-11 weeks after injection. Animals were deeply anesthetized and received sodium pentobarbital in accordance with guidelines for humane euthanasia of animals at the CNPRC. All material injected into rhesus macaques was free of endotoxins (<0.1 EU/mL), and protein purity was confirmed by sodium dodecyl sulphate–polyacrylamide gel electrophoresis (SDS-PAGE). See Supplementary Tables 1 and 2 for route of administration, AAV variants, viral dose, genetic cargo, and duration of expression for each experiment.

#### Pool testing in newborn rhesus macaques

Macaques were perfused with ice-cold RNase-free PBS. At the time of perfusion, one hemisphere of the brain was flash-frozen and the other hemisphere was sectioned into 4 mm coronal blocks and post-fixed in 4% PFA for 48 hours and transferred to Caltech for further processing. For HA staining, we incubated slices with rabbit anti-HA (1:200; Cell Signaling Technology, 3724), performed 3-5 washes with PBS, incubated with donkey anti-rabbit IgG (1:200; Jackson ImmunoResearch, 711-605-152), and washed 3-5 times before mounting. We diluted all antibodies and performed all incubations using PBS supplemented with 0.1% Triton X-100 (Sigma-Aldrich, T8787) and 10% normal donkey serum (Jackson ImmunoResearch, 017-000-121) overnight at room temperature with shaking.

To isolate viral DNA and whole RNA, 100mg slices from brain and liver were homogenized in TRIzol (Life Technologies, 15596) using a BeadBug (Benchmark Scientific, D1036) and total DNA and RNA were recovered according to the manufacturer’s recommended protocol. Recovered DNA was treated with RNase, restriction digested with SmaI, and purified with a Zymo DNA Clean and Concentrator Kit (D4033). Recovered RNA was treated with DNase, and cDNA was generated from the mRNA using Superscript III (Thermo Fisher Scientific, 18080093) and oligo(dT) primers according to the manufacturer’s recommended protocol. We used PCR to amplify the barcode region using 50 ng of viral DNA or cDNA as template. After Zymo DNA purification, we diluted samples 1:100 and further amplified the barcode region using primers to append adapters for Illumina next-generation sequencing. After cleanup, these products were further amplified using NEBNext Dual Index Primers for Illumina sequencing (New England Biolabs, E7600) for ten cycles. We then gel-purified the final PCR products using a 2% low-melting point agarose gel (ThermoFisher Scientific, 16520050). Pool testing enrichment was calculated identically to library enrichment, but is represented in Fig 2b and c on a linear scale.

#### Individual characterization of CAP-Mac in newborn rhesus macaques

Macaques were perfused with PBS and 4% PFA. The brain was sectioned into 4 mm coronal blocks and all tissue was post-fixed in 4% PFA for 3 days before storage in PBS. All tissue was transferred to Caltech for further processing. Brains and liver were sectioned into 100 μm slices using vibratome. Sections of spinal cord were incubated in 30% sucrose overnight and embedded in Optimal Cutting Temperature Compound (Scigen, 4586) and sectioned into 50 μm slices using a cryostat (Leica Biosystems, CM1950). All slices were mounted using Prolong Diamond Antifade and imaged using a confocal microscope. For GFP staining of spinal cord and brain slices from the LP-administered macaque, we incubated slices with chicken anti-GFP (1:500; Aves Bio, GFP-1020), performed 3-5 washes with PBS, incubated with donkey anti-chicken IgY (1:200; Jackson ImmunoResearch, 703-605-155), and washed 3-5 times before mounting. We diluted all antibodies and performed all incubations using PBS supplemented with 0.1% Triton X-100 (Sigma-Aldrich, T8787) and 10% normal donkey serum (Jackson ImmunoResearch, 017-000-121) overnight at room temperature with shaking.

For morphological reconstruction, we sectioned brains into 300 μm sections and incubated them in refractive index matching solution (RIMS)^87^ for 72 hours before mounting on a slide immersed in RIMS. We imaged using a confocal microscope and 25x objective (LD LCI Plan-Apochromat 25x/0.8 Imm Corr DIC) using 100% glycerol as the immersion fluid. We captured tiled Z-stacks (1024x1024 each frame using suggested capture settings) around cells of interest and cropped appropriate fields of view for tracing. Tracing was done in Imaris (Oxford Instruments) using semi-automated and automated methods.

For neuron (NeuN) quantification, slices were stained using anti-NeuN antibody (1:200; Abcam, ab177487) overnight in PBS supplemented with 0.1% Triton X-100 and 10% normal donkey serum. Slices were washed 3-5 times with PBS and incubated overnight in anti-rabbit IgG antibody conjugated with Alexa Fluor 647 (1:200; 711-605-152, Jackson ImmunoResearch) in PBS + 0.1% Triton X-100 + 10% normal donkey serum. After 3-5 washes and mounting using Prolong Diamond Antifade, we obtained z-stacks using a confocal microscope and a 25x objective. We segmented NeuN and XFP-positive cells using custom scripts in Python and Cellpose (https://www.cellpose.org/)^88^.

#### Ex vivo two-photon imaging

Brain slices of sizes suitable for imaging were prepared with a thickness of 400 μm from larger slices using a vibratome and stored in artificial cerebrospinal fluid bubbled with carbogen gas before two-photon imaging, as previously described^89,90^. For testing GCaMP8s responses, electrical stimulation (4-5 V, 80 Hz, 0.3 second duration) with the indicated number of pulses was delivered using an extracellular monopolar electrode placed 100-200 μm away from the neuron imaged. The frame rate of imaging was 30 Hz. Traces of segmented ROIs were plotted as ΔF/F_0_ = (F(t) - F_0_)/F_0_, where F_0_ is defined as the average of all fluorescence value before the electrical stimulation. The rise time was defined as the time required for the rising phase of the signal to reach from 10% of the peak to 90% of the peak. The decay time constant was obtained by fitting sums of exponentials to the decay phase of the signal. The signal-to-noise ratio (SNR) was obtained by dividing the peak amplitude of the signal by the standard deviation of the fluorescence trace before the electrical stimulation.

#### Characterization in adult rhesus macaque slice

One adult rhesus macaque (14 years and 1 month; 10.83 kg) from the Washington National Primate Research Center was planned for routine euthanasia, and the brain was collected as part of the facility’s Tissue Distribution Program. A block of the superior temporal gyrus was sectioned into 300 μm slices and slices were recovered^89^ and cultured on an air-liquid membrane interface^91^ as previously described. Approximately 30 minutes after plating slices, we administered 1-2 μL of AAV (5 x 10^13^ vg/mL of AAV9 or AAV.CAP-Mac packaging either ssCAG-FXN-HA or ssCAG-eGFP). Experiments were performed in biological triplicates for each condition and culture medium was refreshed every 48 hours until tissue collection at 8 days post-transduction. On the day of tissue collection, the slices were imaged to confirm transduction, slices were cut in half, and each half-slice was flash-frozen in a dry ice-ethanol bath. Samples were stored at −20 °C until further processing.

Each half-slice was processed (one each for DNA and RNA recovery). DNA was isolated using the Qiagen DNeasy Blood and Tissue Kit (Qiagen, catalog # 69504) and RNA was recovered using TRIzol (Thermo Fisher Scientific, catalog #15596026) and the PureLink RNA Mini Kit (Thermo Fisher Scientific, catalog # 12183018A). DNA was removed from the RNA sample by modifying the first wash of the PureLink RNA Mini Kit as follows: wash with 350 uL of Wash Buffer 1, then add 80 uL of RNase-Free DNaseI in RDD buffer (Qiagen catalog # 79254) and incubate the column at room temperature for 15 minutes, then wash again with 350 uL of Wash Buffer 1 before proceeding with the protocol. We performed first-strand cDNA synthesis from 400 ng total RNA in 20 uL reactions using Promega GoScript Reverse Transcription Kit (Promega, catalog # A5000).

We then evaluated vector genomes and viral transcripts found in each sample using quantitative qPCR on a Roche Lightcycler II. 100 ng of DNA was used in a 20 uL amplification reaction using TaqMan probes from Thermo Fisher Scientific (EGFP-FAM probe, Assay ID Mr04097229_mr, catalog #4331182; custom genomic reference probe CN2386-2-VIC, Assay ID ARH6DUK, catalog #4448512, designed to target both *Macaca mulatta* and *Macaca nemestrina*).

### Green monkey experiments

All green monkey (*Chlorocebus sabaeus*) procedures were performed at Virscio, Inc. and approved by their IACUC. All monkeys were screened for neutralizing antibodies and confirmed to have < 1:5 titer. At approximately 7-8 months of age (1-1.3 kg), monkeys were dosed intravenously (see Supplementary Table 3 for details). Dose formulations were allowed to equilibrate to approximately room temperature for at least 10 minutes, but no more than 60 minutes prior to dosing. IV dose volumes were based on Day 0 body weights. Animals were sedated with ketamine (8 mg/kg) and xylazine (1.6 mg/kg). The injection area was shaved and prepped with chlorohexidine and 70% isopropyl alcohol, surgically scrubbed prior to insertion of the intravenous catheter. Dosing occurred with a single intravenous infusion of AAV (7.5 x 10^13^ or 7.6 x 10^13^ vg/kg) on Day 0 via a saphenous vein administered using a hand-held infusion device at a target rate of 1 mL/minute. General wellbeing was confirmed twice daily by cage-side observation beginning one week prior to dosing. At the scheduled sacrifice time, monkeys were sedated with ketamine (8-10 mg/kg IM) and euthanized with sodium pentobarbital (100 mg/kg IV to effect). Upon loss of corneal reflex, a transcardiac perfusion (left ventricle) was performed with chilled phosphate buffered saline (PBS) using a peristaltic pump set at a rate of approximately 100 mL/min until the escaping fluid ran clear prior to tissue collection. Cubes of tissue were collected from the left brain hemisphere and various other organs and frozen in the vapor phase of liquid nitrogen for further processing for biodistribution. The right brain hemisphere was removed and cut into ~4 mm coronal slices and post-fixed intact with approximately 20 volumes of 10% neutral-buffered formalin (NBF) for approximately 24 hours at room temperature.

Genomic DNA was extracted from CNS and peripheral tissues using the ThermoFisher MagMax DNA Ultra 2.0 extraction kit (Catalog number: A36570). DNA was assessed for yield by fluorometric quantification with the Qubit dsDNA assay. Approximately 20 ng of DNA was loaded into each 20 μL reaction and plates were run on the BioRad CFX Connect Real-Time PCR Detection System (Catalog number: 1855201). The viral copy number assay was validated for specificity by detection of a single amplified product, sensitivity by assessing the lower limit of detection to be greater than 10 copies per reaction, and linearity by ensuring the standard curve r^2^ was > 0.95. Reactions were assembled in FastStart Universal SYBR Green Master (Rox) (catalogue number: 4913850001). The sequences of the primers were: forward ACGACTTCTTCAAGTCCGCC, reverse TCTTGTAGTTGCCGTCGTCC. The PCR protocol used an initial denaturation step of 95 °C for 180 seconds, followed by 40 cycles of 95 °C for 15 seconds, and 60 °C for 60 seconds, with an imaging step following each 60 °C cycle. A standard curve was generated with linearized plasmid containing the GFP template sequence present in the virus from 1e8-1e0 copies, diluted in naïve untreated macaque DNA samples prepared using an identical kit as the samples in this study to control for matrix effects. Copies of viral DNA were calculated from the standard curve using the equation for the line of the best fit. MOI values were calculated based on the measured total genomic weight of host cell DNA per reaction.

Post fixation, tissues were placed into 10% > 20% > 30% sucrose for 24 hours each at 4 °C then embedded in Optimal Cutting Temperature Compound and stored at −80 °C until cryosectioning. Tissue blocks were brought up to −20 °C in a cryostat before sectioning into 30 μm slices and dry-mounted onto slides after cryosectioning. After sectioning, the slides were left at room temperature overnight to dry. To assist in neuron quantification, we stained sections with the following antibodies and concentrations: rabbit anti-GFP (1:100; Millipore-Sigma, AB3080) and mouse anti-NeuN (1:500; Millipore-Sigma, MAB377). For secondary antibody staining, the following secondary antibodies and concentrations were used: donkey anti-rabbit Alexa Fluor 488 (1:500; Invitrogen, A21206) and donkey anti-mouse Alexa Fluor 647 (1:500; Invitrogen, A31571). All antibodies were diluted with 1X PBS supplemented with 0.25% Triton X-100 (PBST) and 5% normal donkey serum. Primary antibody incubations were left overnight at room temperature. Sections were then washed with PBST. Secondary antibody incubations were 2 hours at room temperature. The sections were washed 3x in PBST. Sections were incubated in DAPI solution (1:10,000; Invitrogen, D1306) at room temperature for 5 minutes, then washed. Sections were coverslipped using Prolong Diamond Antifade.

3 sections per animal were stained and imaged. Each section was imaged in triplicate with each ROI having a total of 9 images. Tissue ROIs were imaged with a Keyence BZ-X800 with the following acquisition parameters: GFP (1/500 s), Cy5 (1 s), DAPI (1/12 s), High Resolution, Z-stack @ 1.2 um pitch. The following brain subregions were imaged frontal, parietal, temporal, occipital cortices, cerebellum, caudate, putamen, and thalamus (medial, ventral lateral, and ventral posterior nuclei). A semi-automated cell counting method was performed via ImageJ for quantification. Using thresholds and particle analysis, we were able to quantify NeuN positive and DAPI positive cells. Using ImageJ’s cell counter, we manually counted GFP-positive and GFP & NeuN double-positive cells.

### Induced pluripotent stem cell (iPSC) experiments

Neuronal cultures were produced by differentiating and maturing iPSC-derived neural progenitor cells with Stemdiff^™^ Forebrain Differentiation and Maturation kits (StemCell #08600, #08605 respectively), according to their manufacturer’s protocols. Neural progenitor cells were produced by differentiation of the foreskin fibroblast-derived iPSC line: ACS^™^-1019 (ATCC# DYS-0100), with Stemdiff^™^ SMADi Neural Induction kits (StemCell l#08581), selection with Stemdiff^™^ Neural Rosette Selection Reagent (StemCell l#05832), and expansion in Stemdiff^™^ Neural Progenitor Media (StemCell l#05833), according to their manufacturer’s protocols. Neurons were matured a minimum of 8 days prior to replating for transduction.

Mature neuronal cultures, seeded 15,000 cells/well in polyornithine and laminin coated black-walled 96 well optical plates, were cultured an additional 4 days prior to transduction. Replicate wells were transduced with virus serially diluted across six orders of magnitude in 90% maturation media and 10% OptiproSFM. 4 days post-transduction, cultures were fixed with 4% paraformaldehyde and counterstained with 1 ug/ml Hoechst 33322. Identification of transduced cells was determined by imaging 60 fields/well, using two channel fluorescence detection (Hoechst at ex386/em440, eGFP ex485/em521) on a CellInsight CX5 HCS Platform. Individual cells were identified by Hoechst detection of their nuclei and applying size and contact constrained ring masks to each cell. Cell transduction was determined by measuring eGFP fluorescence above a threshold level within an individual ring mask. For each population, the percentage of transduced cells was plotted vs the applied dose. Curve-fits and EC_50_ values were determined with a Prism GraphPad [agonist] vs response (three parameter) regression method. To report per-cell eGFP expression efficiencies, the eGFP spot fluorescence intensities were averaged from each ring mask across a minimum of 5,000 cells/well. Curve fits were obtained using the Prism GraphPad Biphasic, X as concentration regression method.

## Supplementary Material

1

## Data Availability

The data that support the findings of this study are available from the corresponding author upon reasonable request. Plasmids used to generate AAV.CAP-Mac will be deposited on Addgene prior to final submission.

## Code availability statement

NeuN quantification was performed using CellPose (https://www.cellpose.org/)^88^ and ImageJ. All next-generation sequencing data was processed using previously-published open-source software from our lab (https://github.com/GradinaruLab/protfarm and https://github.com/GradinaruLab/mCREATE)^16^.
