# Supplementary material for: Intravenous functional gene transfer throughout the brain of non-human primates using AAV
Source: Res Sq. 2023 Jan 13:rs.3.rs-1370972. Preprint. [Version 1] doi: 10.21203/rs.3.rs-1370972/v1 (PMC9928057; doi:10.21203/rs.3.rs-1370972/v1)
Supplement: 1 [file NIHPPrs1370972v1-supplement-1.pdf]

## Supplementary Fig. 1: Generating AAV libraries and choosing variants for further characterization.

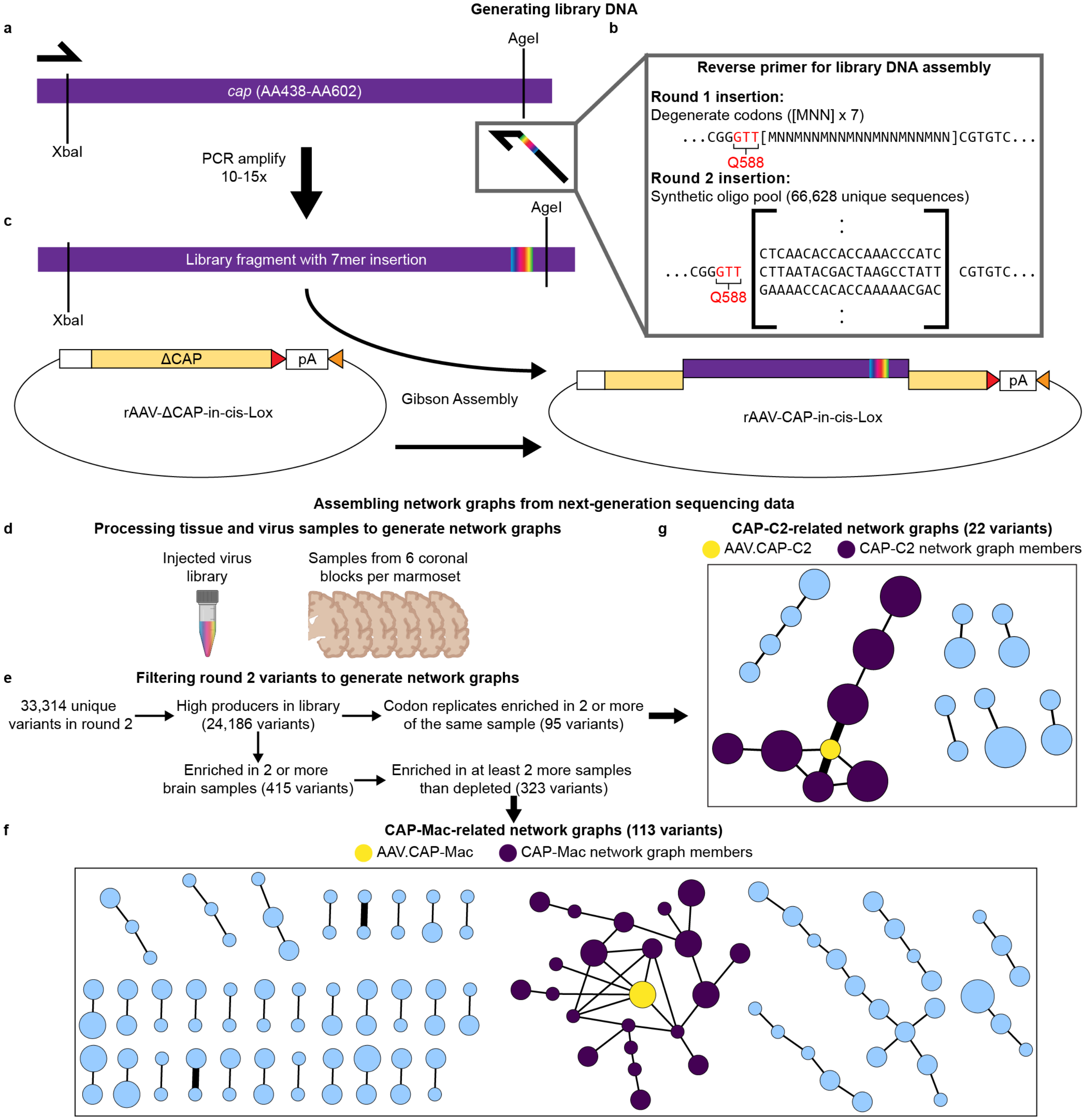


**Supplementary Fig. 1: Generating AAV libraries and choosing variants for further characterization. a**, We introduced diversity into the AAV9 *cap* genome using a reverse primer with a 21-nucleotide insertion after Q588. The reverse primer is used to generate a PCR fragment approximately spanning the XbaI and AgeI section of the modified *cap* gene (approximately AA438 to AA602). **b**, For DNA assembly for round 1 selections, the reverse primer contains 21 degenerate codons ([MNN] x 7]). For round 2 selections, we used a synthetic oligo pool to specify each 21 bp sequence that we insert into *cap*. **c**, The PCR-amplified fragment contains homologous regions that overlap with the rAAV-ΔCAP-in-cis-Lox digested plasmid, and the two fragments are assembled using Gibson assembly to create the final assembled library DNA. **d-g**, Process to assemble network graphs from next-generation sequencing data. While our previous CREATE-based selections have relied on Cre-transgenic mouse lines to increase selection stringency, Cre-transgenic marmosets are currently unavailable, and we were unable to confer this additional selective pressure during selections. We reasoned that through this clustering analysis, we could efficiently and productively sample variants from our selections to (1) limit the number of animals used for individual characterization and (2) partially overcome the absence of the selective pressure provided by Cre-transgenic mice in CREATE. **d**, To generate network graphs, we processed the injected virus library and sampled from each of the 6 brain sections from each animal. **e**, From our next-generation

sequencing data, we calculated library enrichment scores and filtered the variants using two separate criteria. **f**, **g**, Network graphs for AAV.CAP-Mac (**f**) and AAV.CAP-C2 (**g**). CAP-Mac and CAP-C2 were both chosen because they were the most interconnected nodes within their respective networks. Each node represents a unique variant recovered from the round 2 selection and each edge represents pairwise reverse Hamming distance ≥ 3.

## Supplementary Fig. 2: Administering AAV via intrathecal lumbar puncture.

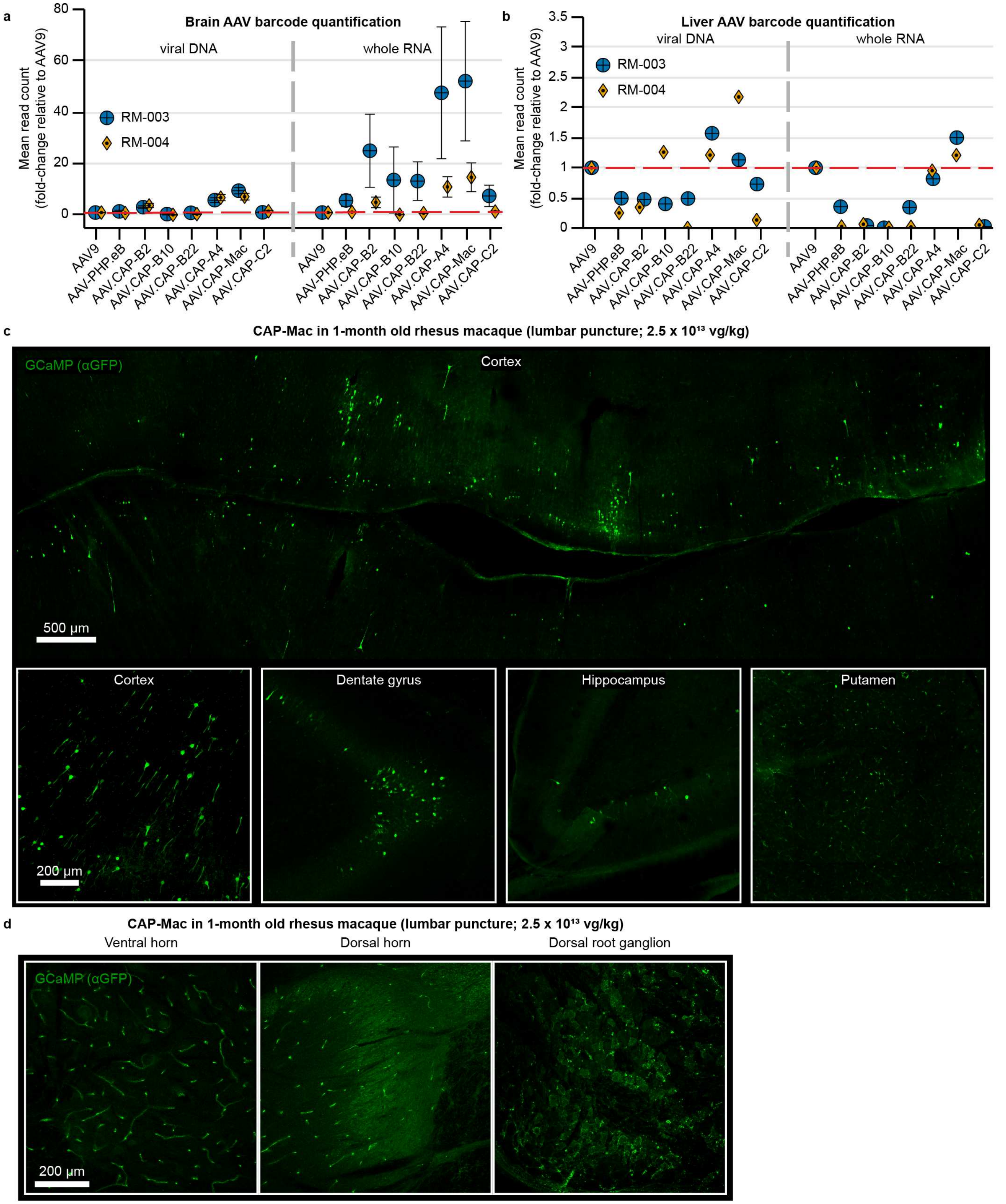


**Supplementary Fig. 2: Administering AAV via intrathecal lumbar puncture.** **a**, **b**, Barcode quantification in viral DNA and whole RNA from brain (**a**) and liver (**b**) of neonate rhesus macaques treated with a capsid pool via intrathecal lumbar puncture. Mean ± s.e.m. shown. **c**, **d**, CAG-GCaMP7s expression in brain (**c**) and spinal cord (**d**) after intrathecal lumbar puncture administration using AAV.CAP-Mac.

**Supplementary Fig. 3: CAG-XFP expression in non-brain tissue of Old World primates treated with AAV.**

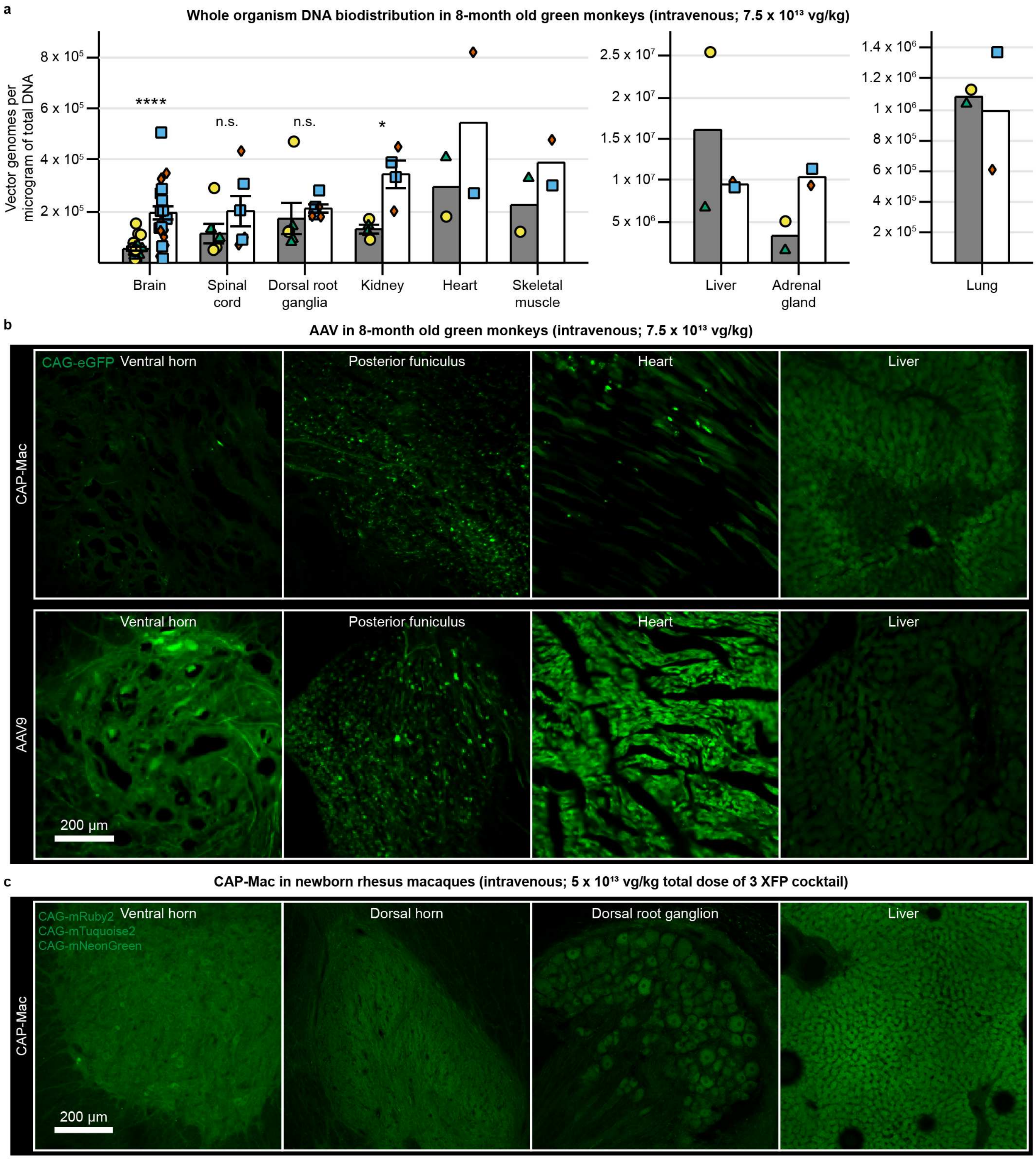


**Supplementary Fig. 3: CAG-XFP expression in non-brain tissue of Old World primates treated with AAV. a**, Vector genomes per microgram of total DNA in green monkeys treated with AAV9 or CAP-Mac, expressed as fold-change relative to mean AAV9. Each data point represents measured vector genomes per microgram of total DNA in a section of tissue from each region and monkey. Mean ± s.e.m. shown. Two-tailed Welch's t-test (*P<0.05, ****P<0.0001). **b**, CAG-eGFP expression in the spinal cord, heart, and liver of green monkeys after intravenous expression of either CAP-Mac (top) or AAV9 (bottom). **c**, CAG-XFP expression in the spinal cord, dorsal root ganglia, and liver of newborn rhesus macaque after intravenous administration of CAP-Mac packaging a cocktail of 3 CAG-XFPs. XFPs are pseudocolored identically.

**Supplementary Fig. 4: Group-level analyses of two-photon calcium imaging in rhesus macaque slice.**

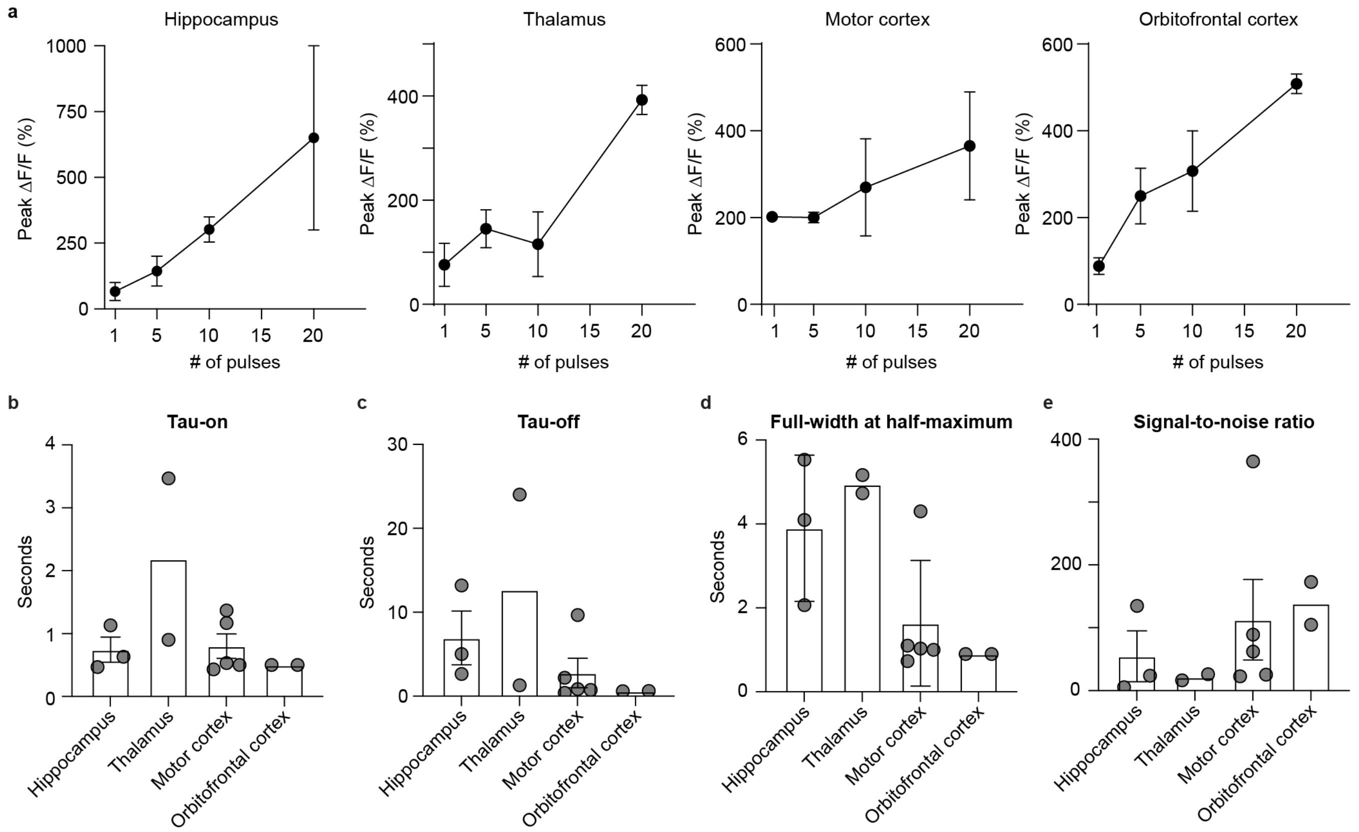


**Supplementary Fig. 4: Group-level analyses of two-photon calcium imaging in rhesus macaque slice. a**, Mean Peak $\Delta F/F_0$ evoked by cells from four different brain regions after applying different number of pulses. **b**, Mean rise time of GCaMP8s responses in the four brain regions. Rise time is defined as time taken for the response to rise from 10% to 90% of the peak of the amplitude. **c**, Mean decay time constant of GCaMP8s responses in the four brain regions. Decay time constant was obtained by fitting sums of exponentials to the decay phase of the traces. **d**, Mean full width at half maximum (FWHM) of GCaMP8s responses in the four brain regions. **e**, Mean signal-to-noise ratio (SNR) of GCaMP8s responses in the four brain regions. SNR is defined as the peak amplitude divided by the standard deviation of the fluorescence signal before the electrical stimulation. Data is plotted as mean ± s.e.m.

**Supplementary Fig. 5: Tropism in rodents and utilizing mice as a model organism for cargo validation.**

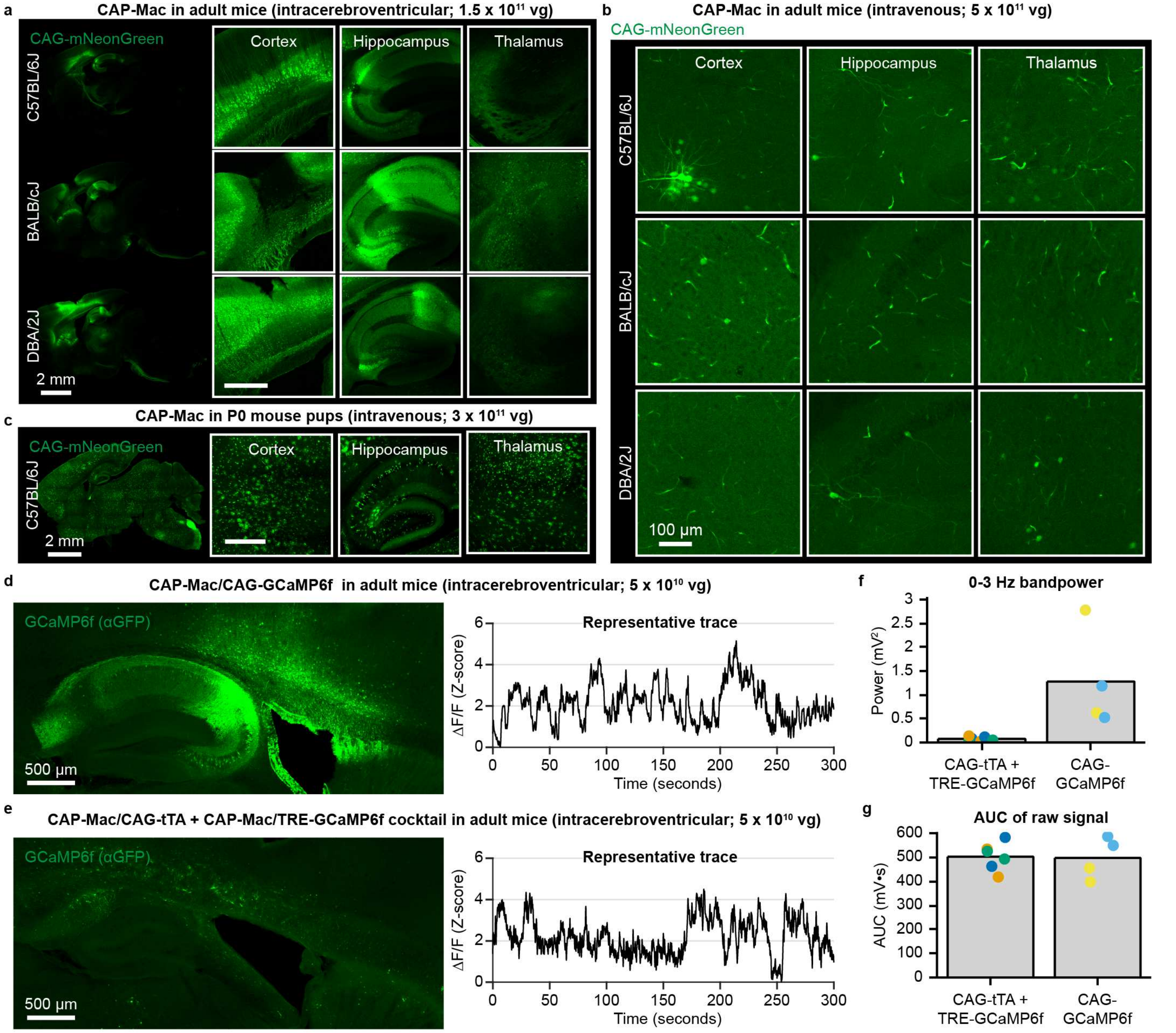


**Supplementary Fig. 5: Tropism in rodents and utilizing mice as a model organism for cargo validation. a**, CAP-Mac after intracerebroventricular (ICV) administration in adult mice primarily transduces neurons. **b**, CAP-Mac after intravenous administration in C57BL/6J, BALB/cJ, and DBA/2J adult mice primarily transduces vasculature. **c**, CAP-Mac in P0 C57BL/6J pups after intravenous administration transduces various cell-types, including neurons, astrocytes, and vasculature. **d-g,** Given the neuronal tropism of CAP-Mac via ICV administration, we validated GCaMP cargo in mice prior to non-human primate experiments. **d**, **e**, GCaMP protein expression and representative ΔF/F traces in mice after delivering CAG-GCaMP6f (**d**) or a CAG-tTA/TRE-GCaMP6f cocktail using CAP-Mac. **f**, **g**, To determine cargo to move forward with, we found that 0-3 Hz bandpower (two-tailed Welch's t-test, P=0.105) (**f**), but not area under the curve (AUC; **g;** two-tailed Welch's t-test, P=0.626) was indicative of cargo performance.

## Supplementary Fig. 6: Tropism in adult common marmoset.

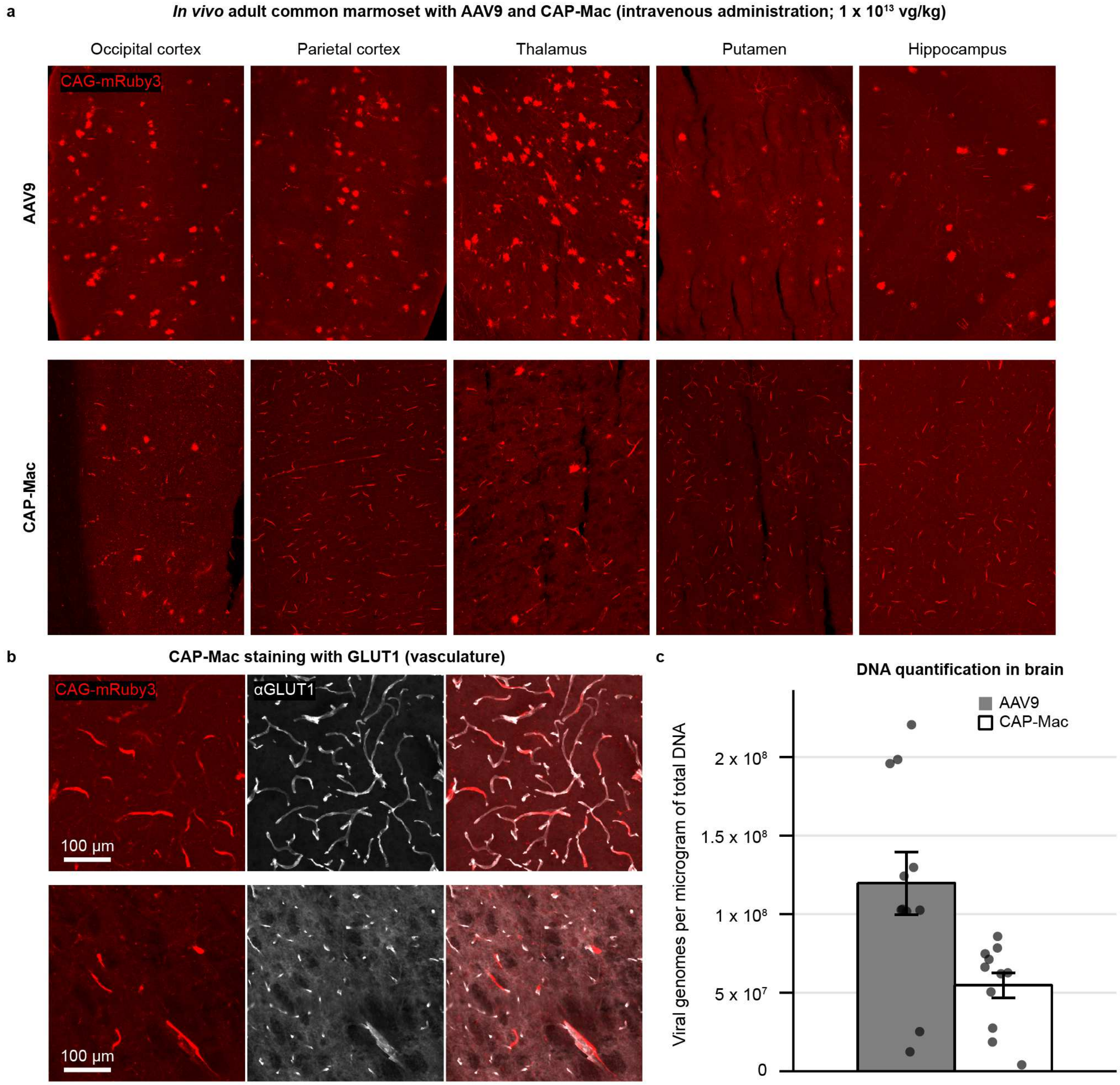


**Supplementary Fig. 6: Tropism in adult common marmoset. a**, AAV9 and CAP-Mac tropism in two adult marmosets *in vivo* (3.8- and 5.8-years-old). **b**, CAP-Mac is biased primarily towards GLUT1+ cells (vasculature). **c**, Recovered viral genomes in two adult marmosets. Mean ± s.e.m. shown. Two-tailed Welch's t-test, P=0.00981.

**Supplementary Fig. 7: Liver function tests in newborn rhesus macaques.**

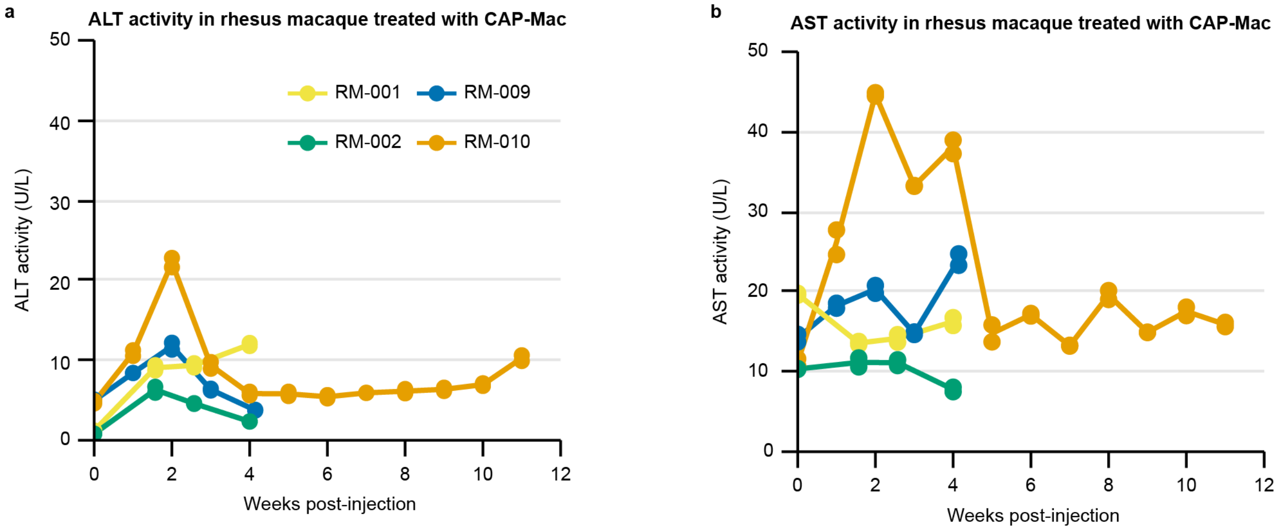


**Supplementary Fig. 7: Liver function tests in newborn rhesus macaques. a**, **b**, Liver function tests show no abnormal signs of adverse liver functionality, as measured by alanine transaminase (ALT; **a**) and aspartate transaminase (AST; **b**) activity.

# Supplementary Files

This is a list of supplementary files associated with this preprint. Click to download.

- rev2supplementarytables.pdf
